# The *Porphyromonas gingivalis* Ferric Uptake Regulator Orthologue Binds Hemin and Regulates Hemin-Responsive Biofilm Development

**DOI:** 10.1371/journal.pone.0111168

**Published:** 2014-11-06

**Authors:** Catherine A. Butler, Stuart G. Dashper, Lianyi Zhang, Christine A. Seers, Helen L. Mitchell, Deanne V. Catmull, Michelle D. Glew, Jacqueline E. Heath, Yan Tan, Hasnah S. G. Khan, Eric C. Reynolds

**Affiliations:** Oral Health Cooperative Research Centre, Melbourne Dental School, Bio21 Institute, The University of Melbourne, Victoria, Australia; Montana State University, United States of America

## Abstract

*Porphyromonas gingivalis* is a Gram-negative pathogen associated with the biofilm-mediated disease chronic periodontitis. *P. gingivalis* biofilm formation is dependent on environmental heme for which *P. gingivalis* has an obligate requirement as it is unable to synthesize protoporphyrin IX *de novo*, hence *P. gingivalis* transports iron and heme liberated from the human host. Homeostasis of a variety of transition metal ions is often mediated in Gram-negative bacteria at the transcriptional level by members of the Ferric Uptake Regulator (Fur) superfamily. *P. gingivalis* has a single predicted Fur superfamily orthologue which we have designated Har (heme associated regulator). Recombinant Har formed dimers in the presence of Zn^2+^ and bound one hemin molecule per monomer with high affinity (K_d_ of 0.23 µM). The binding of hemin resulted in conformational changes of Zn(II)Har and residue ^97^Cys was involved in hemin binding as part of a predicted -^97^C-^98^P-^99^L- hemin binding motif. The expression of 35 genes was down-regulated and 9 up-regulated in a Har mutant (ECR455) relative to wild-type. Twenty six of the down-regulated genes were previously found to be up-regulated in *P. gingivalis* grown as a biofilm and 11 were up-regulated under hemin limitation. A truncated Zn(II)Har bound the promoter region of *dnaA* (PGN_0001), one of the up-regulated genes in the ECR455 mutant. This binding decreased as hemin concentration increased which was consistent with gene expression being regulated by hemin availability. ECR455 formed significantly less biofilm than the wild-type and unlike wild-type biofilm formation was independent of hemin availability. *P. gingivalis* possesses a hemin-binding Fur orthologue that regulates hemin-dependent biofilm formation.

## Introduction

Chronic periodontitis is an inflammatory disease of the supporting tissues of the teeth associated with specific bacteria in a biofilm and is a major cause of tooth loss [1]. *Porphyromonas gingivalis* is considered to be a principal pathogen in chronic periodontitis due to its close association with the disease in humans and its virulence in animal models [1]–[5]. *P. gingivalis* and other oral bacterial species exist *in vivo* as a polymicrobial biofilm called subgingival plaque accreted onto the surface of the tooth root. *P. gingivalis* has recently been described as a ‘keystone pathogen’ that manipulates the host response to allow proliferation of the subgingival plaque community to produce dysbiosis and disease progression [6]. Sessile *P. gingivalis* cells release antigens, toxins and hydrolytic enzymes such as proteinases into the surrounding tissue that stimulate and dysregulate the host immune response causing tissue destruction [7].

Like most bacteria, *P. gingivalis* has an essential growth requirement for iron but unlike most bacteria *P. gingivalis* cannot synthesize protoporphyrin IX, a porphyrin derivative that complexes ferrous iron (Fe^2+^) to form heme, a cofactor used with various enzymes and in electron transport systems [8]. Thus *P. gingivalis* must acquire protoporphyrin IX from the environment, which may explain the reported preferential utilisation of heme as an iron source by this bacterium [9]. *P. gingivalis* also utilises manganese especially for protection from oxidative stress and intracellular survival in host cells [10], [11]. Vascular disruption and bleeding are characteristics of periodontitis, providing an iron/heme rich environment for bacterial growth. However, *P. gingivalis* would also be exposed to low iron/heme environments and oxidative stress during colonization and periods of disease quiescence. In response to this dynamic environment, *P. gingivalis* must tightly regulate iron homeostasis gene expression to survive. We have characterised the *P. gingivalis* W50 response to hemin-limitation in continuous culture, using proteomic and transcriptomic approaches that identified 160 genes and 70 proteins that are differentially regulated by hemin availability [12]. We have also demonstrated the importance of ferrous iron uptake in *P. gingivalis* W50 using the ferrous iron transporter mutant W50FB1, which has half the iron content of the wild-type, and was avirulent in an animal model of disease [13].

Iron homeostasis is mediated in most Gram-negative bacteria and in Gram-positive bacteria with low GC content by the transcriptional repressor protein Fur, using ferrous iron as co-factor [14], [15]. During iron-rich conditions Fur binds intracellular Fe^2+^, acquiring a conformation able to bind target DNA sequences, known as Fur boxes that are found overlapping the promoters of Fur-regulated genes and thereby inhibits transcription of these genes. When iron is scarce, the equilibrium shifts to release Fe^2+^, Fur dissociates from the Fur box and allows access to RNA polymerase and the genes are expressed [16]. Fur is dimeric, and in addition to the labile Fe^2+^ binding site (S2) it also binds zinc in a structurally important site (S1) and can have a further metal binding site per monomer (S3) [17], [18]. The molecular mechanisms of transcriptional control by Fur appear to be shared by many bacterial species, as Fur orthologues from numerous species are able to complement an *E. coli fur* mutant [16]. The genes regulated by *E. coli* Fur encode proteins that are not only involved in iron uptake [19], but also in cellular processes such as defence against oxygen radicals [20], metabolic pathways [21], chemotaxis [22], and the production of toxins and other virulence factors [23], [24]. Iron responsive Fur is the best characterized member of a larger Fur superfamily, with Fur-like proteins responding to manganese (Mur), zinc (Zur), nickel (Nur), hydrogen peroxide (PerR) and iron via heme (Irr) [18].

Characterisation of the *P. gingivalis* Fur orthologue we have designated Har (**h**eme **a**ssociated **r**egulator) showed a hemin and iron-binding transcriptional regulator that plays a role in hemin-responsive biofilm development. We have further demonstrated a relationship between Har, hemin availability and biofilm development with the Har regulon overlapping previously identified *P. gingivalis* hemin-responsive and biofilm adaptation regulons [12]. *P. gingivalis* is unique as an iron-dependent Gram-negative bacterium with a single Fur superfamily orthologue, Har that regulates hemin-dependent biofilm formation.

## Materials and Methods

### Bacterial strains and culture conditions

The bacterial strains and plasmids used in this study are listed in Table 1. *P. gingivalis* ATCC 33277 was obtained from the culture collection of the Oral Health CRC, Melbourne Dental School, The University of Melbourne. Strains ECR455 and ECR475 were derived from ATCC 33277 during this study. *P. gingivalis* strains were routinely maintained on Horse Blood Agar (HBA) plates (HBA; 40 g/L Blood Agar Base No. 2 (Oxoid), 100 mL/L Defibrinated Horse Blood (Equicell, Bayles, Victoria, Australia) containing 5 µg/mL vitamin K and antibiotic selection of 10 µg/mL erythromycin or 5 µg/mL ampicillin where appropriate. Batch cultures of all *P. gingivalis* strains were grown without antibiotics in Brain Heart Infusion (BHI; Oxoid) or Mycoplasma Basal Broth (MBB; Becton, Dickinson and Company BBL) supplemented with 0.5 g/L cysteine, 5 µg/mL hemin and 5 µg/mL vitamin K. All *P. gingivalis* cultures were incubated anaerobically at 37°C in a MACS MG500 anaerobic workstation (Don Whitley Scientific). *P. gingivalis* ATCC 33277 and ECR455 were grown in continuous culture in Bioflo 110 biofermentors (New Brunswick Scientific) as previously described [12], with a 400 mL working volume in BHI supplemented with 0.5 g/L cysteine, 5 µg/mL hemin and 5 µg/mL vitamin K.

**Table 1:** Bacterial strains and plasmids used in this study.

Bacterial strain or plasmid	Description^a^	Reference or source
**Strains**		
*Escherichia coli*		
Alpha-Select Gold	F^-^ *deo*R *end*A1 *rec*A1 *rel*A1 *gyr*A96 *hsd*R17(r_k_^-^, m_k_^+^) *sup*E44 *thi*-1 *pho*A Δ(*lac*ZYA-*arg*F)U169 Φ80*lac*ZΔM15 λ^-^	Bioline
BL21(DE3)	F– *omp*T *hsd*SB(rB–, mB–) *gal dcm* (DE3)	Bioline
*Porphyromonas gingivalis*		
ATCC 33277	Wild-type	Oral Health CRC
ECR455	*P. gingivalis* 33277 *har*::*ermF*, Em^r^	This study
ECR475	ECR455 *ermF*::*har cepA*, Ap^r^	This study
**Plasmids**		
pBluescript II	Cloning vector; Ap^r^	Stratagene
pGEM-TEasy	Cloning vector linearized with T overhangs for ligation of PCR products generated with A overhangs by Taq polymerase; Ap^r^	Promega
pGEX-4T-Har	pGEX-4T-1 containing *har* and an engineered Factor Xa cleavage site, for cleavage of the expressed full-length Har from an N-terminal GST tag.	This study
pGEX-4T-Har150	pGEX-4T-1 containing a 3′ shortened *har* gene and an engineered Factor Xa cleavage site, for cleavage of the expressed C-terminally truncated Har150 from an N-terminal GST tag.	This study
pGEX-4T-HarC97A	pGEX-4T-Har which had the TGT codon for Cys97 mutated to GCA for Ala.	This study
pVA2198	*E. coli*-*Bacteroides* shuttle vector carrying *ermF*-*ermAM* cassette; Em^r^	[76]
pHarSOE1-4	Recombination cassette for the deletion of *har* from *P. gingivalis* 33277, cloned blunt into the SmaI site of pBluescript II; Ap^r^ in *E. coli*	This study
pEC474	*cepA* in pBR322; Ap^r^	[77]
pHarComp	Recombination cassette for the insertion of *har* into *P. gingivalis* ECR455, cloned via A-overhangs into pGEM-T Easy; Ap^r^	This study

a Ap^r^, ampicillin resistant; Em^r^, erythromycin resistant.

### DNA analysis and manipulations

Oligonucleotide primers used in this study are listed in Table 2. Genomic DNA from *P. gingivalis* strains was prepared using the DNeasy Blood and Tissue kit (Qiagen) and plasmid DNA was extracted from *E. coli* strains using the QIAprep spin miniprep kit (Qiagen). The Herculase II DNA Polymerase (Stratagene) and Platinum Taq DNA Polymerase High Fidelity (Invitrogen) were used according to manufacturer's instructions in PCR reactions. SOE PCRs (gene splicing by overlap extension PCRs) were performed essentially as previously described [25]. PCR products were purified using the NucleoSpin Extract II purification kit (Macherey Nagel) according to manufacturer's instructions. Ligations were transformed into *E. coli* alpha-select gold competent cells (Bioline) by heat shock according to manufacturer's instructions. DNA was sequenced by Applied Genetics Diagnostics, The University of Melbourne.

**Table 2:** Oligonucleotides used in this study.

Oligonucleotide Primers	Sequence (5′-3′)^a^
**Recombinant expression of GST-Har and variants**	
HarNterm	CGGAATTCATCGAAGGTCGTATGATAGTCACATCA
HarCterm	CGCTCGAGCCATATCGGATCAATGTTATATGTCT
Har150_Nterm	CCGCGTGGATCCATGATAGTCACATCACTG
Har150_Cterm	CGTGGATCCCGGGTTACTGCTTCTTCCTGCATTT
HarC97A SDM	GCTTCATTTGCAGAGCAGGCACCGCTGCTTTTCTGTACC
**DNA target for EMSA**	
PGN_0001_240bp_For	GGTGTTGATAACTCGGTCGCGCCTT
PGN_0001Rev	CTAAAAAAATATCGTTTTGAGAGCAGT
**Construction of *har* mutant**	
PGN_1502-Fwd	ATGTCGCCTTCCGAGGCTAT
ErmF-PGN_1502-Rev	GCAATAGCGGAAGCTATCGGTTATCTTTTCGATCCATTCTTGC
PGN_1502-ErmF-Fwd	AGAATGGATCGAAAAGATAACCGATAGCTTCCGCTATTGC
Term-ErmF-Rev	GTCTTTCGACTGAGCCTTTCGTTTTAGCATCTAATTTAACTTCAATTCC
Term-Prom-region-Fwd	GCTCAGTCGAAAGACTGGGCCTTTCGTTTTACGGAGTGAAAAAGGAGCCG
PGN_1504-Prom-region-Rev	CAATGTTATATGTCTGTGTTATCTCTCTTTTACATCATATTTTCC
Prom-region-PGN_1504-Fwd	AATATGATGTAAAAGAGAGATAACACAGACATATAACATTGATCC
PGN_1504-Rev	GCAGATATTTTGTAGCCTCCATC
**Construction of *har* complement**	
PGN_1502-Fwd	ATGTCGCCTTCCGAGGCTAT
CepA-Har-Rev	ACTTTCCTTAACTCTTTTGACGTCTTATTTTTTCTTCTTGGGAGCGGCT
Har-CepA-Fwd	AGCCGCTCCCAAGAAGAAAAAATAAGACGTCAAAAGAGTTAAGGAAAGT
ErmF-CepA-Rev	TGTGTAGGTTCTAATTGAAGGACAGACGTCTCAAGTCACCGATAG
CepA-ErmF-Fwd	CTATCGGTGACTTGAGACGTCTGTCCTTCAATTAGAACCTACACA
ErmF-Rev	GATACTGCACTATCAACACACTC
**qRT-PCR**	
PGN_0287 RT Fwd	CCAGCAGCACTTTCCATACAAA
PGN_0287 RT Rev	CCACTGATTACGGCCTCATTT
PGN_0448 RT Fwd	AGTAAAGGGGTAGGGCAACG
PGN_0448 RT Rev	ATCGGATTCGTGTTCCAAAGC
PGN_1296 RT Fwd	CCTGCAAGAGCGTGAAGTTG
PGN_1296 RT Rev	GGATCGGAAAGCCGTATAAGC
PGN_1578 RT Fwd	TGTTGTGGAAAGGAGTGTGG
PGN_1578 RT Rev	AGAAGGAATGAAGTCGGTTGTT
PGN_2083 RT Fwd	GCATTCTTTTCTGGCGTAGCA
PGN_2083 RT Rev	TTTGCGAAACGGCACTCCCT

a Underlined sequence of SOE primers indicates the part of the primer that is complementary to the target sequence, with the remainder of the primer providing complementarity with a second PCR product for splicing.

### Expression and purification of recombinant Har proteins

The full-length *har* gene PGN_1503 (501 bp) was PCR amplified from *P. gingivalis* 33277 using primers HarNterm and HarCterm which had *Eco*RI and *Xho*I restriction sites respectively. The HarNterm primer also encoded a FactorXa cleavage site (IEGR) immediately before the start of the *har* gene. The *har* gene was also amplified with a C-terminal truncation (450 bp) using primers Har150_Nterm and Har150_Cterm which had *Bam*HI and *Sma*I restriction sites respectively. The full length *har* gene and the expression vector pGEX-4T-1 were each digested with *Eco*RI/*Xho*I, whilst the truncated *har* gene and pGEX-4T-1 were each digested with *Bam*HI/*Sma*I, then ligated and transformed into *E. coli* alpha gold (Bioline). The resulting plasmids pGEX-4T-Har and pGEX-4T-Har150, encoding full-length and truncated Har respectively, were transformed into the expression strain *E. coli* BL21(DE3) (Bioline). Site-directed mutagenesis was performed on the pGEX-4T-Har plasmid using the QuikChange Lightning Site Directed Mutagenesis kit (Agilent) and the HarC97A SDM primer as per manufacturer's instructions.

Recombinant *P. gingivalis* Har, truncated Har150 and the site-directed mutant C97A were over-expressed as glutathione S-transferase (GST) fusion proteins with an engineered N-terminal Factor Xa cleavage site. The recombinant genes were expressed in *E. coli* BL21(DE3) by 5 h induction with 200 µM IPTG at 32°C from cell culture OD_600_ around 0.8. After lysis, the GST-Har fusion proteins were applied to GSTrap HP columns (GE Healthcare) at pH 6.2 in 20 mM phosphate binding buffer containing 500 mM NaCl, 1% Triton X-100 and protease inhibitors (Roche). The GST tag was cleaved on column with Factor Xa (GE Healthcare) at pH 8.0 in 50 mM Tris buffer containing 2 mM CaCl_2_ and 150 mM NaCl. Har and its variants bound to a cation exchange HiTrap HP column (GE Healthcare) in 20 mM phosphate buffer at pH 6.8 and were eluted at an ionic strength of 450 mM NaCl. After further purification with size exclusion chromatography, the Har, Har150 and C97A proteins had a purity of over 95%, with a yield of ∼6 mg/L culture.

### Zinc binding by Har

The zinc contents of recombinant Har and variants were determined by electrospray ionization mass spectrometry (ESI-MS) in the presence and absence of formic acid or by inductively coupled plasma mass spectrometry (ICP-MS) of protein solutions treated with Chelex-100 resin and EDTA with or without 8 M urea. Chelex-100 treatment was carried out with a 100 µM protein solution being extensively dialysed at 4°C against 1 g Chelex-100 resin in 50 mM acetate buffer containing 100 mM NaCl and 5 mM DTT at pH 5.0 [26]. EDTA treatment was performed by overnight incubation of a protein solution (100 µM) at 4°C with 50 mM EDTA at pH 8.0 in 10 mM HEPES containing 150 mM NaCl and 5 mM DTT, with or without 8 M urea, followed by EDTA removal with buffer exchange. Lysozyme was used as a control protein known to bind zinc ions very weakly [27]. To characterise Cys involvement in zinc binding, Zn^2+^ released from Har and Har150 by incubation with the Cys oxidising Ellman's reagent, 5,5′-dithiol-2,2′-nitrobenzoic acid (DTNB) [28], [29] was separated by centrifugation through a 3 kDa MWCO filter and determined by ICP-MS.

### Thiol assay

Free sulfhydryl groups in Har were determined in air with DTNB (120 µM final concentration) in sodium phosphate buffer (0.1 M, pH 8.0) [28], [29]. Protein samples (1.3–5.2 µM) were mixed with DTNB and incubated for 15 min before the absorbance at 412 nm was recorded.

### Determination of oligomeric states of Har and variants

The dimerization of Har, Har150 and C97A was determined by analytical size exclusion chromatography performed on a Superdex 75 HR 10/300 column (GE Healthcare).

### Hemin binding by Har

Hemin solutions were prepared freshly by dissolving porcine hemin chloride (≥98% HPLC, Sigma-Aldrich) in 0.1 M NaOH and then diluted into the TBS buffer (50 mM TrisHCl, 150 mM NaCl, pH 8.0). The stock hemin solution was filtered through a 0.22 µm filter unit (Millipore) and kept cold on ice in the dark before use. Concentrations of hemin were determined with the extinction coefficient of 58,400 M^−1^.cm^−1^ on a Cary50 UV-vis spectrometer [30]. Hemin (1 – 2 µM) was incubated with the purified Har and C97A proteins at 0 to 4 protein to hemin molar ratios. The spectra (700 – 250 nm) of these solutions were collected after 1 h incubation. Dissociation constants of hemin binding by Har and C97A were estimated by fitting the absorbance changes at 419 nm from the spectrophotometric titrations using the biochemical analysis program Dynafit [31]. The hemin binding affinity of each protein was estimated from separate titrations using three different hemin concentrations. Lysozyme was used as a negative control in the hemin binding determinations.

### Spectroscopic and affinity estimation of divalent metal cation binding by Har and C97A

Fluorescence spectra were collected on a Cary eclipse fluorescence spectrophotometer (Varian) at room temperature. The Har or C97A mutant concentration was 8.0 µM in 10 mM HEPES buffer (pH 7.0) containing 250 mM NaCl and the reducing agent tris(2-carboxyethyl) phosphine (TCEP) (2 mM). The cation (Fe^2+^ or Mn^2+^) was added at 0–8 molar equivalents to protein. Metal binding affinities were estimated by fitting the titration curves with the biochemical analysis program Dynafit [31].

### Secondary structural analysis

Circular dichroism (CD) data of Har and Har150 in the presence and absence of one equivalent of Fe^2+^ (as (NH_4_)_2_Fe(SO_4_)_2_) or 0.8 equivalents of hemin were acquired from 260 to 190 nm on a Jasco J815 spectropolarimeter [32]. Secondary structures of the proteins were estimated by analysing the CD data using the DichroWeb online server [33], [34].

### Har DNA binding

All EMSA reactions were performed using 50 mM TrisHCl pH 7.0, 40 mM MgCl_2_, 100 mM NaCl, 5 mM DTT and various concentrations of Zn(II)Har150 protein (2.85 – 14 µM), *dnaA* promoter DNA (0.2 µM) or hemin (0 – 140 µM) in a total reaction volume of 20 µL. A negative control EMSA was performed using Zn(II)Har150 and PGN_1308 promoter DNA that is bound by a different *P. gingivalis* transcriptional regulator. FAM-labeled DNA was generated via PCR using 5′FAM-labeled PCR primers (Geneworks). EMSA reactions were incubated 25°C for 2 h then 4°C for 1 h before gently adding DNA loading buffer and loading each EMSA reaction onto a 1% agarose gel, with the wells cast centrally in the gel, in 2 x TA buffer. After electrophoresis DNA was visualized by staining gels in a SYBR Safe DNA gel stain bath (Life Technologies) or FAM fluorescence was visualized with a LAS-3000 Imager. Proteins were visualized by staining gels with SimplyBlue SafeStain (Life Technologies).

### Construction of *P. gingivalis har* mutant and complemented strains

The recombination cassette for deletion of the *har* gene consisted of the final 600 bp of the PGN_1502 gene, the *ermF* gene encoding erythromycin resistance in *P. gingivalis*, followed by a transcriptional terminator then a copy of the promoter region that drives the operon containing PGN_1503, followed by the first 614 bp of the PGN_1504 gene. The truncated PGN_1502 and PGN_1504 genes were used as the sites of homologous recombination with the *P. gingivalis* ATCC 33277 chromosome and would result in replacement of PGN_1503 with *ermF* followed by a transcriptional terminator then the promoter to drive transcription of the remaining genes downstream of PGN_1503 in the operon. This cassette was constructed from four separate PCR products that were spliced together to form the final cassette. The following PCR products were amplified from ATCC 33277 genomic DNA: PGN_1502′ (primers PGN_1502-Fwd and *ErmF*-PGN_1502-Rev), the promoter region (primers Term-Prom-region-Fwd and PGN_1504-Prom-region-Rev) and PGN_1504′ (primers Prom-region-PGN_1504-Fwd and PGN_1504-Rev). The *ermF* gene was amplified from pVA2198 with primers PGN_1502-*ErmF*-Fwd and Term-*ErmF*-Rev. The transcriptional terminator was included in the Term-*ErmF*-Rev (used to amplify *ermF*) and Term-Prom-region-Fwd (used to amplify the promoter region) primers so that when the *ermF* and promoter PCR products were joined by SOE PCR, the terminator sequence would be between them. All PCRs were performed with Herculase II and the final product cloned into the SmaI site of pBluescript to form pHarSOE1-4. The recombination cassette was released from the plasmid with EcoRV/XbaI and 200 ng electroporated into *P. gingivalis* ATCC 33277 in a 0.1 cm gap cuvette at 1.8 kV, 200 Ohms resistance. The resulting mutant was called ECR455.

The recombination cassette for complementation of the *har* mutant consisted of the final 600 bp of the PGN_1502 gene plus the *har* gene PGN_1503, followed by the *cepA* gene encoding ampicillin resistance, then the final 578 bp of the *ermF* gene. The truncated PGN_1502 and *ermF* genes were used as the sites of homologous recombination with the *P. gingivalis* ECR455 chromosome and would result in the insertion of the PGN_1503 gene and the ampicillin resistance gene. This cassette was constructed from three separate PCR products that were spliced together to form the final cassette. PGN_1502′ through to the end of PGN_1503 was amplified from ATCC 33277 chromosome (primers PGN_1502-Fwd and CepA-Har-Rev), the *cepA* gene was amplified from pEC474 (primers Har-CepA-Fwd and ErmF-CepA-Rev) whilst *ermF*′ was amplified from pVA2198 (primers CepA-ErmF-Fwd and ErmF-Rev). All PCRs were performed with Herculase II except for the final SOE PCR which was amplified with Platinum Taq DNA Polymerase High Fidelity, then cloned into pGEM-TEasy to produce pHarComp. This plasmid was electroporated into ECR455 resulting in the *har*-complemented strain, ECR475.

### Western blot analyses of Har expression

Bacterial whole cell lysates or cytoplasmic protein extracts (25 µg) were separated on 4–12% Bis-Tris polyacrylamide gels (Invitrogen) in MES buffer before Western transfer and immunoblotting with rabbit anti-rHar serum diluted 1∶2500.

### Determination of cellular metal content

Three biological replicates of each strain of *P. gingivalis* ATCC 33277, ECR455 and ECR475 were grown in MBB supplemented with 0.5 g/L cysteine, 5 µg/mL hemin and 5 µg/mL vitamin K and cell lysates were prepared as previously described [13]. Measurements were made using an Agilent 7700 series ICP-MS instrument under operating conditions suitable for routine multi-element analysis in Helium Reaction Gas Cell mode.

### Extraction of RNA for transcriptomic analyses

Extraction of total RNA was performed as previously described [12].

### Microarray hybridization and analyses

*Porphyromonas gingivalis* W83 microarray slides version 1 were obtained from the Pathogen Functional Genomics Resource Centre of the J. Craig Venter Institute. cDNA synthesis, labeling and microarray hybridization were all performed as previously described except that 5 µg total RNA was reverse transcribed instead of 10 µg [12]. Paired samples were compared on the same microarray using a two-colour system. A total of 6 paired microarray hybridizations were performed representing 6 biological replicates, where a balanced dye design was used, with the overall analyses including three microarrays where *P. gingivalis* ATCC 33277 samples were labeled with Cy3 and the paired ECR455 samples were labeled with Cy5 and three other microarrays where samples were labeled with the opposite combination of fluorophores. Image analysis was also performed as previously described except that print tip loess normalization was used [12].

### qRT-PCR

Three biological replicates of *P. gingivalis* ATCC 33277, ECR455 and ECR475 were grown in batch culture to an OD_650_ of 1.0 in BHI supplemented with 0.5 g/L cysteine, 5 µg/mL hemin and 5 µg/mL vitamin K. cDNA was generated from RNA isolated from these strains using a NucleoSpin RNA II Total RNA Isolation kit (Macherey-Nagel) and SuperScript III Reverse Transcriptase First-Strand Synthesis SuperMix for qRT-PCR kit with random hexamers (Invitrogen), then qPCR analysis was performed using 0.3 ng cDNA per reaction and Power SYBR Green PCR master mix (Applied Biosystems), all according to manufacturer's instructions. cDNA was quantified relative to standard curves generated by amplification of ATCC 33277 gDNA with the same primer pair used for each cDNA. Primer pairs used are listed in Table 2, with primers for PGN_1296 representing genes that did not change in the microarrays, PGN_0287 and PGN_1578 representing genes that increased in transcription in ECR455, and PGN_0448 and PGN_2083 representing genes that decreased in transcription in ECR455 relative to ATCC 33277.

### Biofilm assays

Parental and mutant strains of *P. gingivalis* grown in BHI supplemented with 0.5 g/L cysteine and 5 µg/mL vitamin K containing either 7 µM hemin (hemin excess) or no added hemin (hemin limitation) for 24 h were diluted in the same medium to a density of 5×10^7^ cfu/mL, and incubated in CultureWell imaging chambers (Invitrogen) for 24 h under anaerobic conditions at 6 rpm on a rocking platform. Four wells were inoculated per strain per experiment. The supernatant was removed and the biofilms washed carefully with 0.85% NaCl to remove any non-adherent cells and stained with the LIVE/DEAD BacLight Bacterial Viability kit (Invitrogen) for 20 min according to the manufacturer's instructions. Wells were then washed a final time with 0.85% NaCl post-staining prior to imaging.

Biofilms were imaged as previously described [35] using a Zeiss LSM 510 META Confocal Laser Scanning Microscope with a C-Apochromat 63x/1.2 numerical aperture, water immersion objective lens fitted with a correction collar. SYTO 9 fluorescence was detected by excitation with a 488 nm Argon Ion laser and emission collected with a 500–550 nm bandpass filter. Propidium Iodide (PI) fluorescence was detected by excitation with a 543 nm Helium Neon laser and emission collected by 560 nm longpass filter. Biofilm images were analysed using the COMSTAT 3D biofilm structure quantifying software [36].

### Statistical analyses

All statistical analyses were performed using Minitab16 statistical software. Following a Levene's test for equal variances, data were analysed using one-way ANOVA with Tukey multiple comparison tests or the Kruskal-Wallis test with pairwise Mann Whitney tests with a Bonferroni correction. The p-value was set at 0.05 and 95% confidence intervals were calculated.

### Microarray data accession number

The microarray data presented in this paper have been entered into the NCBI GEO databank (www.ncbi.nlm.nih.gov/projects/geo) with the accession number GSE37099.

## Results

### *P. gingivalis* Fur orthologue

**Figure 1:**
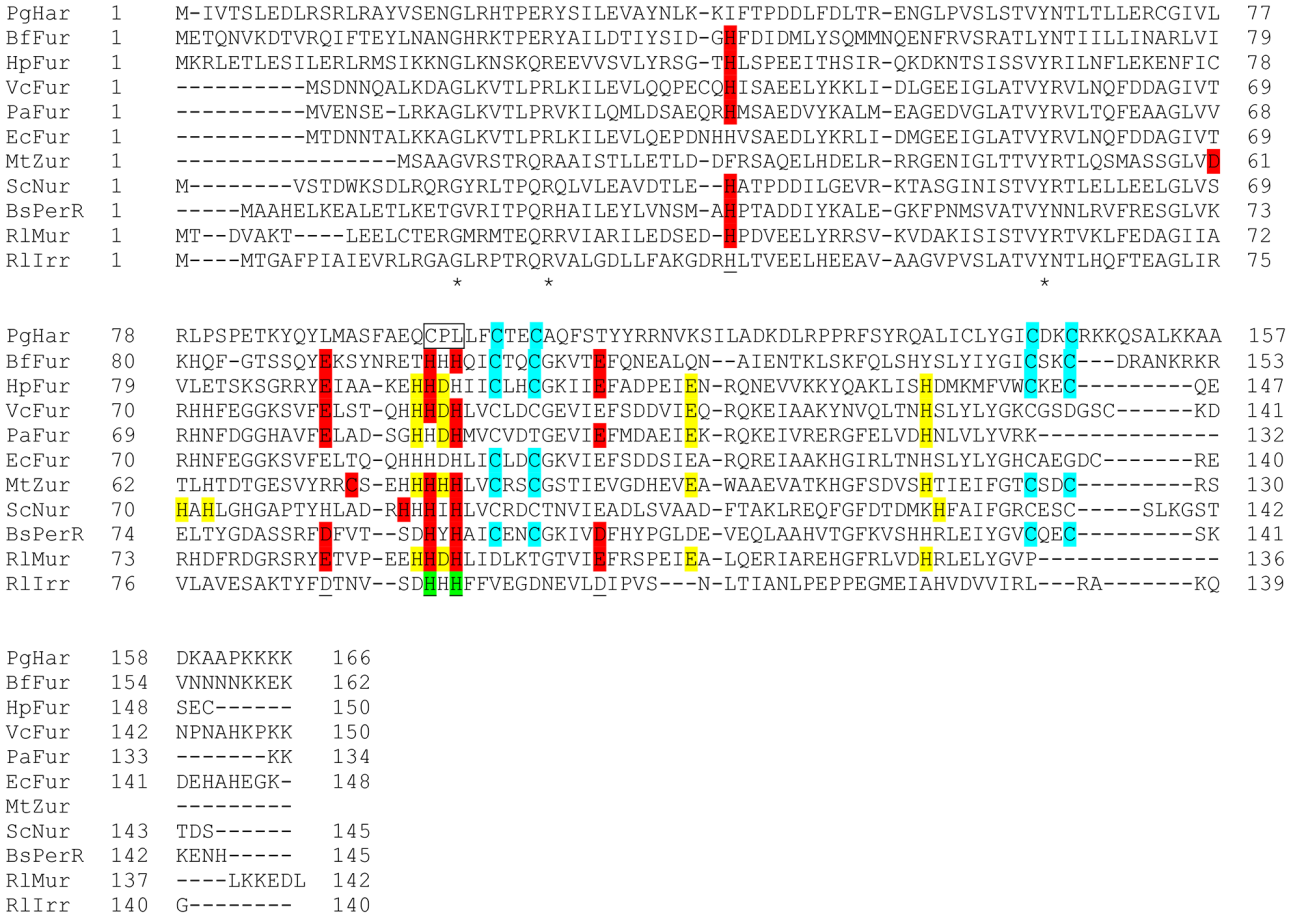
Alignment of Fur family proteins with PGN_1503 (Har) from *P. gingivalis* ATCC 33277. Fur family proteins were aligned using COBALT [75] with *P. gingivalis* Har (PgHar; UniProt B2RKX7). Seven of these proteins have structures in the Protein DataBank: HpFur is the iron responsive Fur from *Helicobacter pylori* (UniProt B9XY52), VcFur is the iron-responsive Fur from *Vibrio cholerae* (UniProt P0C6C8), PaFur is the iron-responsive Fur from *Pseudomonas aeruginosa* (UniProt Q03456), EcFur is the iron-responsive Fur from *Escherichia coli* (UniProt P0A9A9), MtZur is the zinc-responsive FurB from *Mycobacterium tuberculosis* (UniProt O05839), ScNur is the nickel-responsive Nur from *Streptomyces coelicolor* (UniProt Q9K4F8) and BsPerR is the peroxide-responsive PerR from *Bacillus subtilis* (UniProt P71086). The other three Fur family proteins are BfFur, an iron-responsive Fur from *Bacteroides fragilis* NCTC9343 (UniProt Q64QR6), RlMur and RlIrr, the manganese-responsive Mur (UniProt Q1MMB4) and the iron response regulator Irr (UniProt Q1MN49) respectively, from *Rhizobium leguminosarum* bv. *viciae* (strain 3841). * indicates identical amino acids in all 11 proteins. Shading indicates residues experimentally confirmed to be involved in the three distinct metal binding sites: S1 in blue; S2 in red, S3 in yellow as reported in Dian *et al*. [17], except for those in PgHar, BfFur and RlMur which were inferred by similarity to the other sequences. The five residues underlined in RlIrr show the amino acids that would make up S2 and although Irr has been shown to bind metals *in vitro*, the metal binding site is still unknown. The principal heme binding site of RlIrr is the HxH motif shaded green [47], which is part of the S2 motif. The putative heme regulatory motif of PgHar is boxed.

The *P. gingivalis* ATCC 33277 genome sequence contains a single predicted Fur orthologue encoded by PGN_1503 [37], which we have designated Har, for **H**eme **a**ssociated **r**egulator. Strikingly, the predicted pI is high at 9.47 due to the presence of numerous lysine and arginine residues, particularly in the lysine-rich C-terminal tail of 20 residues. The predicted amino acid sequence of *P. gingivalis* PGN_1503 was aligned with FurA from *Bacteroides fragilis* NCTC9343 (BfFur) which is the closest related species to *P. gingivalis* that has an experimentally determined Fur orthologue (Fig. 1) [38]. Har and BfFur share 27% identity and 85% similarity and both have C-terminal tails rich in lysine and arginine residues. Seven other members of the Fur family for which there is structural data were also aligned with PGN_1503 as well as two other representatives of the Fur superfamily, Mur and Irr, from *Rhizobium leguminosarum* (Fig. 1). None of these other Fur family members have the highly positively charged C-terminal tail that is found in Har and BfFur. Har contains the dual -C-X-X-C- motifs involved in binding zinc in the S1 structural site which is found in some but not all Fur family members (Fig. 1). The S2 site is the Fur metal sensory site and metallation of this site is essential for specific DNA binding. The S2 site is conserved in Fur superfamily proteins [17] and can be inferred in all sequences in the alignment except Har (Fig. 1). A predicted heme regulatory motif (HRM) [39] -^97^C-^98^P-^99^L- which RlIrr uses for hemin binding (Fig. 1) was identified in the *P. gingivalis* Har sequence in place of the -H-X-H- S2 motif. Furthermore the S3 site, a supplementary metal binding site that strengthens DNA-binding affinity of the Fur protein [17], was not detectable in the Har sequence. *P. gingivalis* Har and two variants, Har150 which was truncated by 16 amino acids at the C-terminus to remove the lysine rich tail and C97A where Cys97 was substituted with Ala to mutate the proposed hemin binding motif, were then expressed and purified as recombinant proteins from *E. coli* for characterisation.

### Characterisation of zinc binding by recombinant Har and C97A

**Figure 2:**
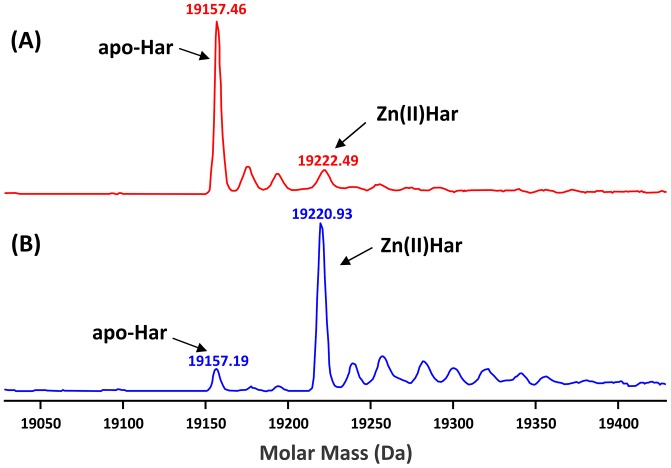
Zinc binding by recombinant Har. Purified Har (5 µM) was buffer exchanged into ammonium acetate (10 mM) via extensive dialysis at 4°C and subjected to ESI-MS analysis on a Quadropole-Time of Flight mass spectrometer (Agilent) in the positive mode with a fragmentor voltage of 200-300 V and a skimmer voltage of 65 V at a flow rate of 500 µL/h for direct syringe infusion delivery to the electrospray probe in the presence (**A**) and absence (**B**) of 0.1% v/v formic acid in the mobile phase. The average molar masses were obtained by application of a deconvolution algorithm to the recorded spectra and were calibrated with horse heart myoglobin (16951.5 Da).

Purified *P. gingivalis* Har and C97A were subjected to ESI-MS analysis in ammonium acetate in the presence of 0.1% v/v formic acid. Har showed a major peak with a molar mass of 19157.46 Da that is consistent with the theoretical mass of apo-Har, 19157.20 Da (Fig. 2). In contrast, in the absence of formic acid the major peak was at 19220.93. This difference in mass corresponds to the atomic mass of zinc minus two protons suggesting that one zinc ion was bound to the protein monomer (Fig. 2). ESI-MS analysis also confirmed the identity of C97A with the measured molar mass of 19125.8 Da consistent with the theoretical mass of 19125.3 Da (data not shown). ICP-MS analysis of Har and C97A confirmed that a single zinc ion was bound to each Har or C97A monomer (Table 3). EDTA could not remove the zinc ions from the proteins however when Har was treated with EDTA under denaturing conditions (8 M urea), negligible levels of zinc were detected after buffer exchange to remove the EDTA and urea (Table 3). As removal of zinc required denaturation of Har the zinc loaded forms (Zn(II)Har and Zn(II)C97A) were used for characterisation studies.

**Table 3:** Zinc content of purified recombinant Har and C97A with and without metal chelator treatment.

Protein sample	Har				C97A	Lysozyme
	Untreated	Treated with 50 mM EDTA	Treated with Chelex-100 resin	Treated with 50 mM EDTA in 8 M urea	Treated with 50 mM EDTA	
Protein concentration (µM)	23.2	23.3	22.9	21.4	14.2	25.5
Zn bound (µM)	22.0	22.0	21.2	0.1	13.4	nd^a^
Zn/Har molar ratio	0.95	0.94	0.93	0.005	0.94	-

Protein solutions were treated with the strong metal chelator Chelex-100 at pH 5.0 or EDTA with or without 8 M urea at pH 8.0. After removal of the metal chelators and urea, the zinc ions in the protein solutions were determined by ICP-MS, with lysozyme as a negative control protein.

a not detected.

### Free thiol assay and release of zinc from Har/Har150 by DTNB

Ellman's reagent detected seven (experimentally 6.6–6.8) free sulfhydryl (-SH) groups in each monomer of Zn(II)Har and Zn(II)Har150 under oxidative conditions, suggesting that all cysteine residues in both proteins exist in a reduced form. This indicates that the protein structure allows all the free thiol groups in the seven cysteine residues to be readily accessible to DTNB but, interestingly, not to be oxidised to disulfide by air. Therefore, neither intramolecular nor intermolecular disulfide bonds were formed in the proteins even in the presence of oxygen.

ICP-MS detected over 0.8 equivalents of zinc in the filtrates of both Zn(II)Har and Zn(II)Har150 in a concentrator of 3 kDa MWCO after incubation with DTNB for 15 min but no released zinc could be detected in the filtrate when in the absence of DTNB (Table 4). DTNB therefore was able to release the bound zinc from Har/Har150, indicating a zinc binding site with cysteines being involved as ligands, which was disabled by oxidation with DTNB. ICP-MS analyses of Har150 treated with DTNB also showed one zinc ion binding stoichiometry of the protein (Table 4), thus truncation of the C-terminal lysine rich tail did not affect the zinc binding ability of Har.

**Table 4:** Zinc ions released from Zn(II)Har and Zn(II)Har150 by the cysteine oxidising reagent DTNB.

	Without DTNB		With DTNB	
Protein sample	Zn(II)Har	Zn(II)Har150	Zn(II)Har	Zn(II)Har150
Protein conc. (µM) a	13.21	7.31	13.21	7.31
Zn in filtrate (µM)	nd c	nd	11.39	5.88
Zn/P b	-	-	0.86	0.81

Zinc content in the filtrates of Zn(II)Har and Zn(II)Har150 after 15 min incubation of the proteins with and without DTNB (160 µM) in 100 mM sodium phosphate pH 8.0 as determined by ICP-MS.

a before centrifugation through the 3 kDa MWCO filter.

b the ratio of zinc content in the filtrate to the protein concentration before centrifugation.

c not detected.

### Oligomerisation states of Zn(II)Har, Zn(II)C97A and Zn(II)Har150

**Figure 3:**
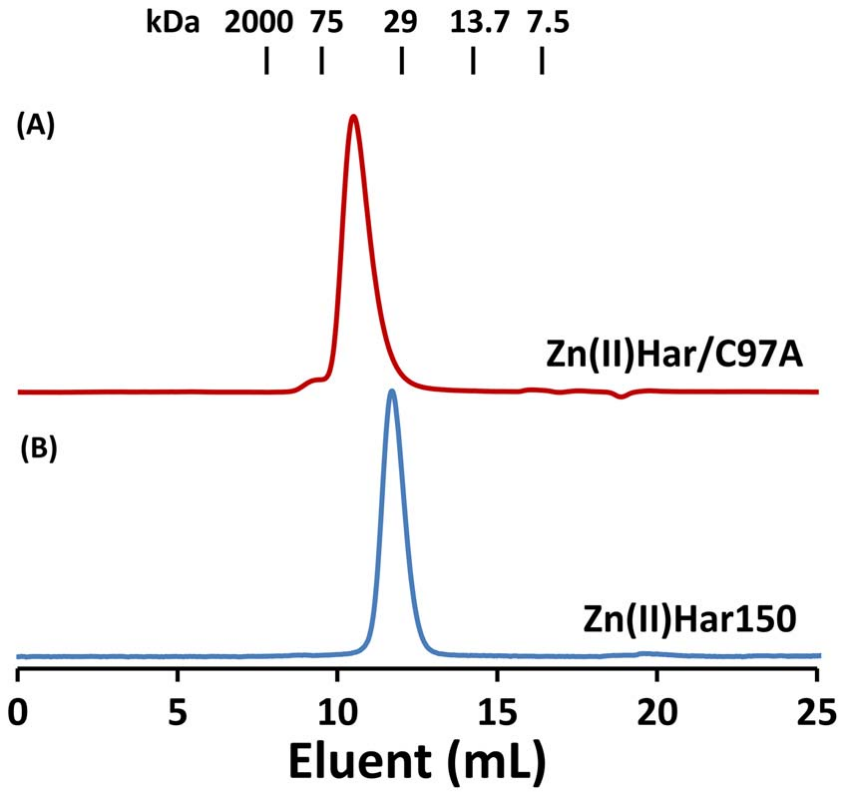
Dimerization of Zn(II)Har, Zn(II)C97A and Zn(II)Har150. Representative elution profiles of Zn(II)Har/Zn(II)C97A (**A**) and Zn(II)Har150 (**B**) from a Superdex 75 analytical gel filtration column at 4°C in an AKTA FPLC Chromatographic System (GE Healthcare). Proteins (100 µg) were applied to the column pre-equilibrated with 500 mM NaCl containing buffers (20 mM) at pH 6.0 (MES), pH 7.0 (HEPES), 7.4 (KPi) and 8.5 (borate). The molar masses were calculated against a calibration curve of retention volumes (*Ve*) of the protein standards, which are indicated at the top of the chart. The elution profiles at pH 7.0 are presented.

Size exclusion chromatography of Zn(II)Har, Zn(II)C97A and Zn(II)Har50 in reducing or non-reducing environments showed a single major peak in each elution profile with an apparent molar mass of 41.0 kDa which corresponded to dimeric Har or C97A with two zinc ions, or 35.0 kDa which corresponded to dimeric Zn(II)Har150 (Fig. 3). The Zn(II)Har homodimer was stable over the pH range 6.0–8.5 and no variation in the elution profile of the dimer was found at 4°C and ambient temperature (22°C) suggesting a stable dimeric form of Zn(II)Har (data not shown). Since all the seven thiol groups are unpaired, such dimerizaton was not caused by intermolecular disulfide formation.

### Hemin binding by Zn(II)Har and Zn(II)C97A

**Figure 4:**
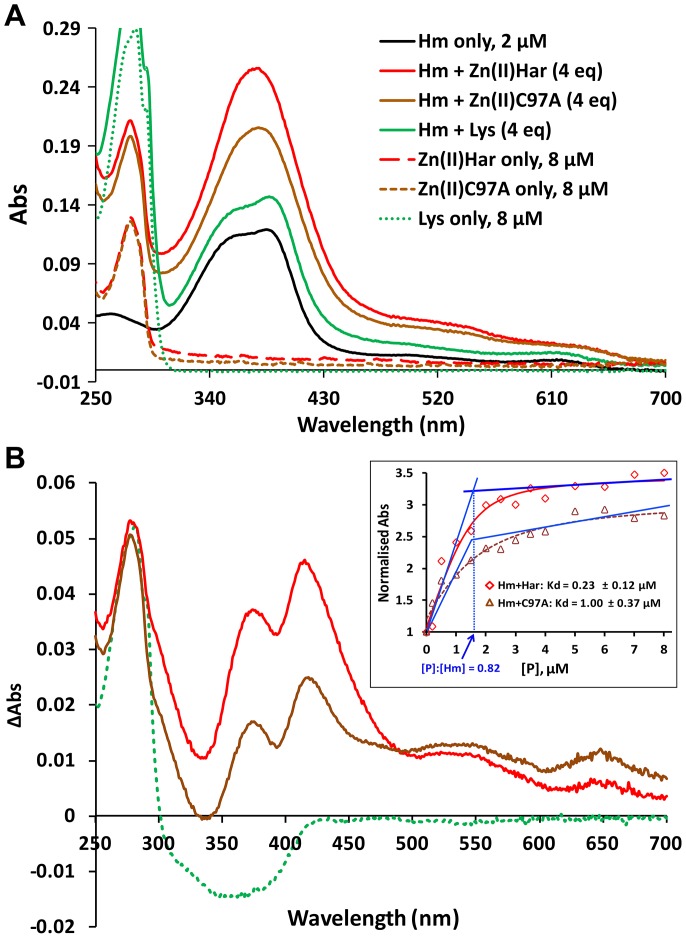
Spectrometric determination of hemin binding by Zn(II)Har and Zn(II)C97A. Hemin (Hm, 1–2 µM) in TBS was incubated with Zn(II)Har and Zn(II)C97A at protein to hemin molar ratios of 0∶1 to 4∶1 for 1 h and solution spectra collected on a Cary 50 UV-visible spectrometer (Varian). Lysozyme (Lys) was used as a negative control (green lines). (**A**) Absorption spectra of 2 µM free hemin, 8 µM Zn(II)Har or Zn(II)C97A, and hemin plus four equivalents of Zn(II)Har or Zn(II)C97A. Based on the hemin binding affinities of Zn(II)Har and Zn(II)C97A estimated in (B), at the starting protein:hemin molar ratio of 4∶1, free hemin in the equilibrium solution was 3.8% and 15.3% of the total hemin after reaction with Zn(II)Har and Zn(II)C97A, respectively. (**B**) Spectra of 1∶1 protein to hemin (2 µM) molar ratio are presented as a subtraction from the spectrum of hemin only (red line for Zn(II)Har, brown line for Zn(II)C97A, green line for lysozyme). The hemin binding affinity of the protein was estimated by fitting the absorbance changes at 419 nm for Zn(II)Har and Zn(II)C97A against protein concentrations (inset) using the biochemical analysis program Dynafit [31]. Inset: Fitted titration curves, apparent dissociation constants (K_d_) and the titration data point sets of the normalised absorbance at 419 nm for Zn(II)Har and Zn(II)C97A. Estimation of binding stoichiometry is shown in blue. P: protein.

Addition of Zn(II)Har or Zn(II)C97A to hemin induced a solution spectrum change that indicated the formation of a complex between hemin and each protein. UV-visible spectra of the Zn(II)Har-hemin and Zn(II)C97A-hemin complexes showed a blue shift of the typical hemin absorption peak at 388 nm in the near UV range by ∼16 nm (372 nm; Fig. 4A) consistent with hemin binding to HRMs [39] and indicating both proteins had affinity for hemin. Difference absorption spectra of the Zn(II)Har-hemin and Zn(II)C97A-hemin complexes showed a second absorption maxima at ∼420 nm (Fig. 4B). The intensities of the Soret maxima for Zn(II)C97A were reduced with respect to that of the wild-type spectrum. Addition of lysozyme to hemin resulted in no obvious change to the hemin spectrum. Titration of a hemin solution with Zn(II)Har or Zn(II)C97A showed that Zn(II)Har bound one hemin molecule per monomer with high affinity (K_d_ of 0.23±0.12 µM), and Zn(II)C97A had a four-fold lower affinity for hemin with a K_d_ of 1.00±0.37 µM (Fig. 4B, inset).

### Divalent metal cation binding by Zn(II)Har

**Figure 5:**
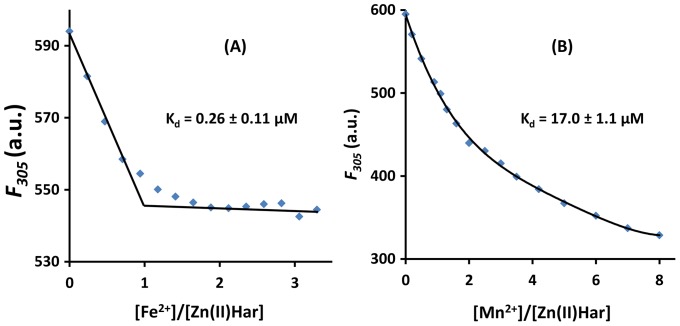
Divalent metal cation binding by Zn(II )**Har.** Fluorescence spectroscopic titration of Zn(II)Har with ferrous ions and manganese ions in HEPES (5 mM, pH 7.0) containing 250 mM NaCl in the presence of TCEP (2 mM). Change in fluorescence emission intensity of Zn(II)Har (8 µM) at 305 nm upon addition of 0 – 3.3 molar equivalent Fe^2+^ (**A**) and 0 – 8 molar equivalent Mn^2+^ (**B**), with each set of presented data being averaged from three individual titrations. Apparent dissociation constants (K_d_) were estimated by fitting the titration data using the biochemical analysis program Dynafit [31]. λ_ex_ = 275 nm.

There are ten tyrosine residues in Zn(II)Har which act as fluorophores allowing fluorescence to be used as a probe to determine divalent cation binding activity. The protein exhibited intrinsic fluorescence to a maximum intensity at 305 nm upon excitation at 275 nm. Fluorescence spectroscopic titration of the protein at micromolar concentration with ferrous ions showed a linear decrease in fluorescence intensity at 305 nm up to one molar equivalent of ferrous ions. Further addition of ferrous ions essentially did not alter the fluorescence intensity, suggesting a 1∶1 molar ratio of iron to protein monomer stoichiometry with a K_d_ of 0.26 µM (Fig. 5). In contrast Zn(II)C97A interacted with Fe^2+^ non-specifically, resulting in a small fluorescence change with no end point on addition of the metal ion under the same conditions (data not shown). Fluorescence titration of Zn(II)Har with Mn^2+^ showed a nonlinear decrease in fluorescence intensity indicating a relatively lower binding affinity with a K_d_ of 17 µM (Fig. 5).

### Secondary structure analyses

Addition of Fe^2+^ or hemin to Zn(II)Har caused significant changes to secondary structure (Table 5). Addition of hemin (0.8 eq) to Zn(II)Har in PBS caused a decrease in the α-helical content from 46% to 36% and β-strand content from 20% to 16% with an increase in unordered content. Addition of Fe^2+^ (1.0 eq) to Zn(II)Har in 10 mM HEPES buffered saline (75 mM NaCl) at pH 7.0 also resulted in a significant change in secondary structure by decreasing the α-helical content from 44% to 35%. While the β-strand content remained essentially the same, the content of turns decreased and unordered structure increased. Similarly, the presence of Fe^2+^ or hemin had a significant effect on the secondary structure of Zn(II)Har150. The addition of either Fe^2+^ or hemin to Zn(II)Har150 caused a significant decrease in its α-helical content, accompanied by an increase in percentage of β-strands and turns (Table 5).

**Table 5:** Secondary structure of Zn(II)Har and Zn(II)Har150 in the absence or presence of Fe^2+^ or hemin.

	α-helix	β-strand	Turns	Unordered	Total
Zn(II)Har only	0.44	0.13	0.19	0.23	0.99
+ Fe^2+^ (1 eq)	0.35	0.15	0.12	0.37	0.99
Zn(II)Har only	0.46	0.20	0.14	0.20	1
+ Hemin (0.8 eq)	0.36	0.16	0.14	0.35	1.01
Zn(II)Har150 only	0.34	0.23	0.21	0.22	1
+Fe^2+^ (1 eq)	0.27	0.28	0.26	0.20	1.01
Zn(II)Har150 only	0.33	0.18	0.23	0.26	1
+ Hemin (0.8 eq)	0.17	0.26	0.26	0.31	1

Secondary structures were estimated by DichroWeb [33], [34] analyses of the CD data of the proteins (5 µM) at 20°C in the absence or presence of Fe^2+^ (1 eq) and hemin (0.8 eq) in 10 mM HEPES reducing buffer (75 mM NaCl, 0.5 mM TCEP, pH 7.0) and 10 mM PBS (75 mM NaCl, pH 7.4), respectively.

### Har DNA binding

**Figure 6:**
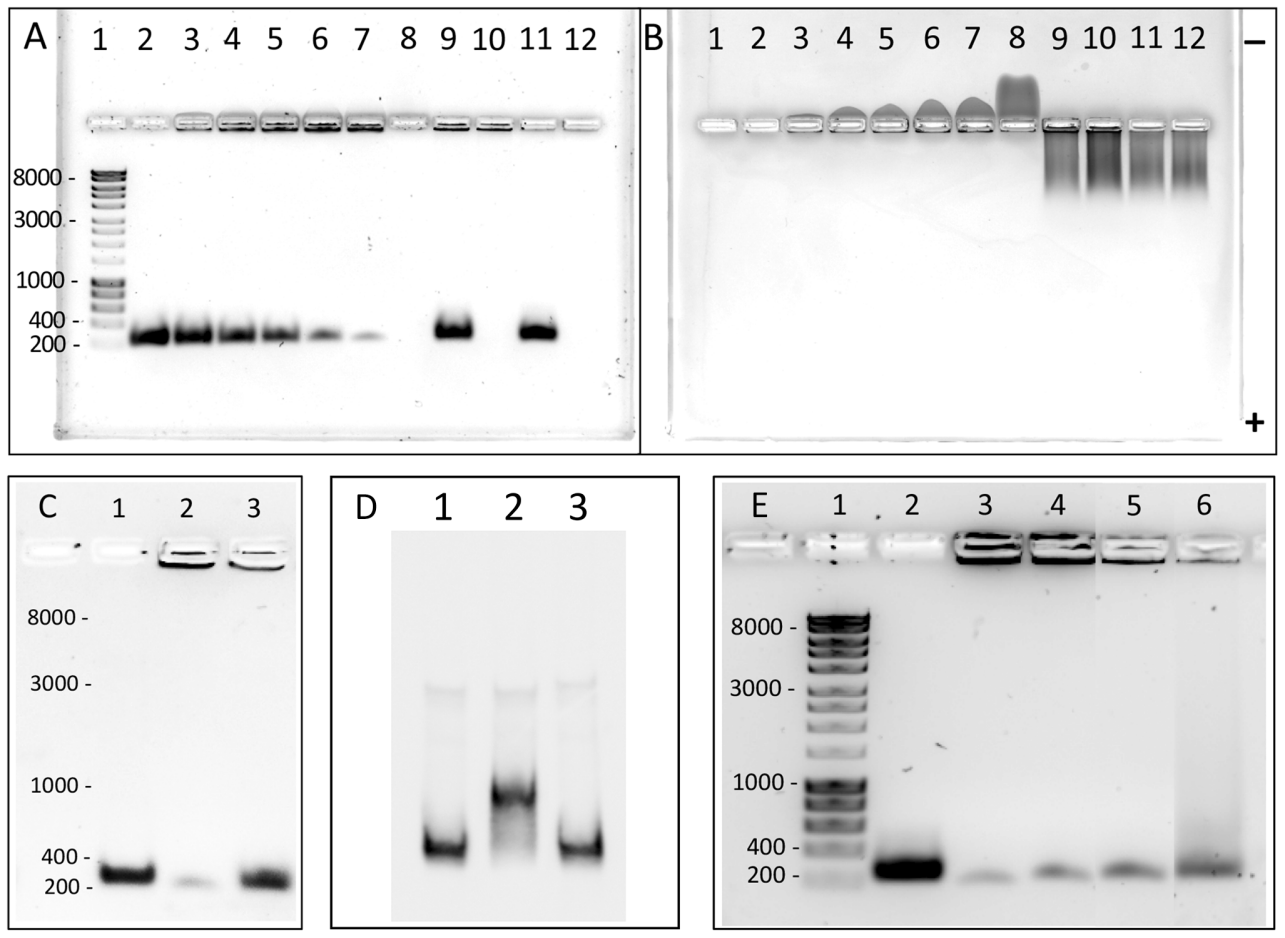
EMSA of Zn(II)Har150 binding to DNA. (A). Agarose gel electrophoresis stained with SYBR Safe DNA gel stain (Life Technologies) for visualizing DNA. Lane 1, HyperladderI (Bioline) DNA size markers in bp. Lanes 2–7, 9 & 11 all contain 500 ng (0.2 µM) of a 240 bp PCR product encompassing the 33277 *dnaA* promoter sequence (PGN_0001). Additionally, lane 3 has 1 µg (2.8 µM) Zn(II)Har150; lane 4, 2 µg (5.6 µM) Zn(II)Har150; lane 5, 3 µg (8.4 µM) Zn(II)Har150; lane 6, 4 µg (11.2 µM) Zn(II)Har150 and lane 7, 5 µg (14 µM) Zn(II)Har150. Lane 2 contains DNA only whereas Lane 8 contains 5 µg (14 µM) Zn(II)Har150 only. Lanes 9 – 12 contain the negative control protein for DNA binding, BSA, where there is 5 µg BSA in lanes 9 & 10, and 3 µg BSA in lanes 11 & 12. (B). Agarose gel electrophoresis stained with SimplyBlue SafeStain (Life Technologies) for visualizing protein following DNA visualisation. Lanes are as described in (A). The position of the anode (+) and cathode (-) are noted. (C). EMSA competition experiment where an excess of unlabeled *dnaA* promoter DNA (1250 ng) competed with 250 ng (0.1 µM) FAM-labeled *dnaA* promoter DNA for binding to 3 µg (8.4 µM) Zn(II)Har150 (lane 3). Lane 1 contains 250 ng FAM-labeled DNA only, whereas lane 2 contains 250 ng FAM-labeled DNA bound to 3 µg Zn(II)Har150. Visualised is the fluorescence of the FAM-labeled DNA after agarose gel electrophoresis (D) EMSA experiment where the promoter-containing DNA of PGN_1308 (lanes 1–3) was shifted by its cognate transcriptional repressor (lane 2) but not by Zn(II)Har150 (lane 3). (E). Inhibition of Zn(II)Har150 DNA binding by hemin. The addition of increasing concentrations of hemin (lane 3, 0 µM; lane 4, 14 µM; lane 5, 70 µM; lane 6, 140 µM) to a constant amount of DNA (500 ng, lanes 2–6) and Zn(II)Har150 (14 µM, lanes 3–6) resulted in increasing inhibition of DNA binding by Zn(II)Har150. Lane 1, HyperladderI (Bioline) DNA size markers in bp. Agarose gel electrophoresis stained with SYBR Safe DNA gel stain (Life Technologies) for visualizing DNA.

Zn(II)Har150 without its lysine-rich tail was used for EMSA analyses to minimize non-specific DNA interactions as it has a lower predicted pI of 8.98 compared with Zn(II)Har (pI of 9.47). The promoter region of *dnaA* (PGN_0001) was used as the Zn(II)Har binding target because *dnaA* has been identified from the microarray results of the current study (*vide infra*) as being negatively regulated by Har. The EMSAs were run on agarose gels with the wells cast centrally to enable electrophoresis of both protein and DNA which are oppositely charged [40]. Zn(II)Har150 bound with the promoter region of *dnaA* forming a Zn(II)Har150-DNA complex preventing the DNA from entering the gel under the applied electric field. The negative control BSA which does not bind DNA did not affect the migration of the *dnaA* promoter region (Fig. 6A). The positively charged Zn(II)Har150 migrated towards the cathode (-) but this movement was retarded in the presence of the specific DNA due to the formation of the Zn(II)Har150-DNA complex (Fig. 6B). Unlabeled specific DNA competed for binding of Zn(II)Har150 to the FAM-labeled specific DNA (Fig. 6C), however non-specific DNA at a similar concentration to the specific DNA did not compete for binding indicating that the binding of Zn(II)Har150 was specific for the *dnaA* promoter. Zn(II)Har150 did not bind to the promoter region of PGN_1308 a DNA target for a different transcriptional regulator of *P. gingivalis* (PgMntR), indicating that Zn(II)Har150 does not bind DNA non-specifically (Fig. 6D). Increasing concentrations of hemin resulted in increasing dissociation of the Zn(II)Har150-DNA complex showing that the binding of Zn(II)Har150 to the *dnaA* promoter region was specifically inhibited by hemin at molar excess concentrations (Fig. 6E).

### Construction of *P. gingivalis har* mutant and complemented strains

**Figure 7:**
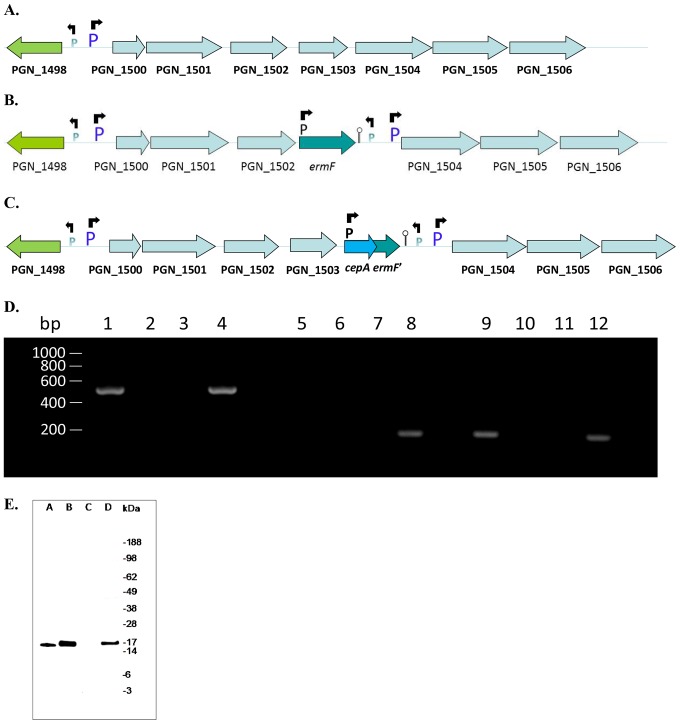
Genomic arrangement of *P. gingivalis* ATCC 33277 in (A) the wild-type strain, (B) *har* mutant strain ECR455 and (C) *har* complemented strain ECR475. ‘P’ denotes promoter positions, the arrows above ‘P’ denote the direction of transcription whilst the stem loop following *ermF* indicates a Rho-independent transcriptional terminator. Not drawn to scale. **(D) RT-PCR analysis of *PGN_1504* and *PGN_1503* (*har*).** Reverse transcription of ECR455 and ECR475 RNA was performed using random hexamers. PCR was then performed using oligonucleotide primers specific for *PGN_1504* (lanes 1–4) or *PGN_1503* (*har*) (lanes 5–8 and 9–12). The templates used for PCR were: reverse transcribed ECR455 RNA (lanes 1 and 5), reverse transcribed ECR475 RNA (lane 9), RNA that was not reverse transcribed (lanes 2, 6 and 10), no template (lanes 3, 7 and 11) and *P. gingivalis* ATCC 33277 genomic DNA (lanes 4, 8 and 12). PGN_1504 transcript was detected in the *har* mutant ECR455 (lane 1), whilst PGN_1503 (*har*) transcript was not detected in the *har* mutant strain ECR455 (lane 5), but was detected in the *har* complemented strain ECR475 (lane 9). **(E) Western blot detection of Har expression in *P. gingivalis* 33277, ECR455 and ECR475.** Cytoplasmic protein extracts (25 µg) from *P. gingivalis* strains 33277 (B), ECR455 (C), ECR475 (D) and 5 ng purified Har (A) were separated on a 4–12% Bis-Tris polyacrylamide gel (Invitrogen) before Western transfer and blotting with anti-rHar sera. Har protein was detected in the 33277 wild-type and ECR475 complement, but not the ECR455 mutant strain.

RT-PCR analysis showed that the *P. gingivalis har* gene is in the midst of an operon (data not shown), thus *har* was deleted such that there was minimal effect on the transcription of genes downstream of *har* (Fig. 7). A recombination cassette was designed where *har* was replaced by *ermF* followed by a strong Rho-independent transcriptional terminator, then a copy of the intergenic region containing the *har* operon promoter. The genes (PGN_1502 and PGN_1504) on either side of *har* were used as the flanking DNA for homologous recombination of the cassette with the *P. gingivalis* chromosome (Fig. 7). Deletion of PGN_1503 from ATCC 33277 to produce strain ECR455 was confirmed by Southern blot and PCR analyses (data not shown). Reverse transcription-PCR showed no *har* transcript, but amplification of PGN_1504 cDNA confirmed transcription of the genes downstream of the deleted *har* gene (Fig. 7).

Complementation of the *har* deletion involved the insertion of the *har* ORF back into position downstream of PGN_1502 followed by *cepA* that was inserted into the *ermF* gene. PGN_1502 and part of the *ermF* sequences were used as flanking DNA for homologous recombination of the cassette into the *har* deletion mutant ECR455 (Fig. 7). The resulting strain ECR475 was shown to have the correct chromosomal arrangement by PCR and the *har* gene amplified from the chromosome was sequenced and found to be correct (data not shown). Furthermore RT-PCR showed transcription of the complemented *har* gene (Fig. 7). Western blot analysis using rHar antisera showed that *P. gingivalis* ATCC 33277 and ECR475 whole cell lysates contained Har whilst the ECR455 *har* deletion mutant did not (Fig. 7).

### Cellular metal content

The cellular metal content of three biological replicate cell lysates of *P. gingivalis* ATCC 33277, ECR455 and ECR475 was determined for 34 elements using ICP-MS. No significant differences were identified in the metal contents of ECR455 (Har mutant) relative to both ATCC 33277 and ECR475 (Har complemented) for any of the 34 elements analysed (Table 6).

**Table 6:** Elemental content of *P. gingivalis* ATCC 33277, ECR455 and ECR475 as determined by ICP-MS.

*P. gingivalis*	Fe	Mn	Zn	Ni	Mg
33277	8,514±251^a^	87±6	1,250±152	2.9±1.1	58,476±2,284
ECR455	9,523±199	78±7	1,088±121	4.1±1.5	62,592±1,448
ECR475	9,751±286	93±11	1,680±354	3.3±1.0	60,793±3,388

a All values are presented as pmol/mg cellular dry weight and represent the mean of three biological replicates for each strain. Metal content was statistically analysed using a one-way ANOVA with Tukey multiple comparison tests. In total 34 different elements: Li, B, Na, Mg, Al, P, K, Ca, Ti, V, Cr, Mn, Fe, Co, Ni, Cu, Zn, Ga, Ge, As, Se, Rb, Sr, Zr, Mo, Rh, Ru, Cd, Sn, Sb, Cs, Ba, W and Pb, were measured.

### Transcriptomic analyses of ECR455 versus wild-type

Total RNA was extracted from six biological replicates of *P. gingivalis* ATCC 33277 and ECR455 grown in continuous culture under defined conditions with a fixed generation time of 8.6 h and used in microarray analyses to identify the Har regulon. A total of 44 genes had significantly altered expression in ECR455 (≥1.5 fold change, p<0.05), compared with the wild-type. Nine genes were up-regulated including two operons (Table 7) whereas 35 genes were down-regulated including 10 operons and 16 genes encoding hypothetical proteins (Table 8). No genes encoding known iron homeostasis or storage proteins had altered expression in the *har* mutant. However the gene transcript encoding the hemophore HmuY decreased ∼2-fold and interestingly 11 of the 35 down-regulated genes have previously been shown to have increased expression in *P. gingivalis* grown under hemin-limitation (Table 8) [12], thus suggesting a relationship between Har and hemin availability. A clear relationship between Har and biofilm growth was also seen, as 26 of the 35 down-regulated genes had previously been found to be up-regulated when *P. gingivalis* was grown as a biofilm compared with planktonic growth (Table 8) [41]. Three operons that were down-regulated have been proposed to play roles in aerotolerance (PGN_0527-31), potassium uptake (PGN_2082-3) and efflux of proteins and small molecules (PGN_0446-9). Quantitative real time PCR (qRT-PCR) analysis of five selected genes confirmed the changes in expression showed by the microarray analysis (Table 9).

**Table 7:** Gene transcripts significantly up-regulated in the *P. gingivalis* ECR455 mutant compared with ATCC 33277 wild-type.

PGN_ID^a^^,^ b	JCVI Probe Name	Gene	Annotation	Cellular Role	Fold Change	p-Value
PGN_0001	PG0001	*dnaA*	Chromosomal replication initiator protein DnaA	DNA replication, recombination and repair	1.69	9.3E-10
PGN_0287	PG0176	*mfaI*	MfaI fimbrillin	Cell envelope surface structure	1.81	1.5E-4
PGN_1578	PG0387	*tuf*	Translation elongation factor Tu	Translation factor	1.78	3.0E-3
PGN_1851	PG1921	*rpsE*	30S ribosomal protein S5	Translation – ribosomal protein	1.52	2.5E-3
PGN_1853	PG1923	*rplF*	50S ribosomal protein L6	Translation – ribosomal protein	1.68	1.7E-2
PGN_1858	PG1928	*rplN*	50S ribosomal protein L14	Translation – ribosomal protein	1.72	1.8E-2
PGN_1860	PG1930	*rpmC*	50S ribosomal protein L29	Translation – ribosomal protein	1.51	1.5E-3
PGN_2088	PG2224	*husD*	Hypothetical protein	Unknown, part of operon encoding hemophore HusA	1.77	2.6E-2
PGN_2089	PG2225	*husC*	Transcriptional regulator MarR family	Proposed regulator of HusA hemophore expression	1.68	3.6E-2

a Results are sorted by ascending PGN_ID (locus ID in 33277).

b Predicted operons: PGN_1851-1860; PGN_2088-2089.

**Table 8:** Gene transcripts significantly down-regulated in the *P. gingivalis* ECR455 mutant compared with ATCC 33277 wild-type.

PGN_ID^a^^,^ b	JCVI Probe Name	Gene	Annotation	Cellular Role	Fold Change	p-Value	HL^c^ Fold Change	Biofilm^d^ Fold Change
PGN_0300	PG0192	*ompH-1*	cationic outer membrane protein OmpH	Cell wall/membrane biogenesis	0.57	7.4E-05	1.62	1.64
PGN_0301	PG0193	*ompH-2*	cationic outer membrane protein OmpH	Cell wall/membrane biogenesis	0.63	1.4E-03	1.50	1.74
PGN_0320	PG0215	*-*	hypothetical protein	Unknown	0.66	1.8E-06	1.45	1.87
PGN_0321	PG0216	*-*	hypothetical protein	Unknown	0.66	2.3E-04		1.88
PGN_0400	PG1715	*-*	hypothetical protein	Unknown	0.51	7.5E-06	1.56	2.06
PGN_0444	PG1667	*-*	outer membrane efflux protein PG52	Intracellular trafficking, secretion, and vesicular transport	0.66	1.1E-04		1.06
PGN_0446	PG1665	*-*	putative ABC transporter permease protein	Defence mechanisms	0.61	9.1E-07		
PGN_0447	PG1664	*-*	putative ABC transporter permease protein	Defence mechanisms	0.54	1.8E-06		1.15
PGN_0448	PG1663	*-*	ABC transporter ATP-binding protein	Defence mechanisms	0.47	6.7E-06		1.21
PGN_0449	PG1662	*-*	hypothetical protein	Unknown	0.58	4.1E-07		1.13
PGN_0449_b	PG1661	*-*	hypothetical protein	Unknown	0.50	7.0E-05		
PGN_0451	PG1659	*-*	hypothetical protein	Unknown	0.57	8.1E-05		1.28
PGN_0485	PG1634	*-*	hypothetical protein	Unknown	0.64	4.8E-04	1.60	2.08
PGN_0486	PG1635	*-*	hypothetical protein	Unknown	0.66	1.4E-04	1.51	1.7
PGN_0527	PG1584	*batC*	probable aerotolerance-related exported protein BatC	Unknown	0.66	4.8E-03		1.13
PGN_0529	PG1582	*batA*	aerotolerance-related membrane protein BatA	Coenzyme transport and metabolism	0.60	1.4E-05		1.10
PGN_0531	PG1580	*-*	conserved hypothetical protein	Unknown	0.63	1.4E-05		1.73
PGN_0558	PG1551	*hmuY*	HmuY	Heme binding and transport	0.51	4.8E-03	10.1	1.17
PGN_0968	PG0987	*-*	hypothetical protein	Unknown	0.65	3.0E-02		
PGN_0970	PG0985	*-*	RNA polymerase sigma-70 factor ECF subfamily	Transcription	0.59	3.0E-03		1.08
PGN_1019	PG0928	*-*	response regulator	Signal transduction mechanisms	0.59	2.8E-04		1.64
PGN_1021	PG0926	*-*	hypothetical protein	Unknown	0.59	5.2E-06	1.8	1.64
PGN_1104	PG1314	*aroC*	chorismate synthase	Amino acid transport and metabolism	0.40	2.3E-06		2.19
PGN_1105	PG1315	*slyD*	peptidyl-prolyl cis-trans isomerase SlyD, FKBP-type	Posttranslational modification, protein turnover, chaperones	0.57	1.9E-02	1.47	1.49
PGN_1106	PG1316	*-*	hypothetical protein	Unknown	0.53	9.1E-07		1.57
PGN_1107	PG1317	*-*	hypothetical protein	Unknown	0.66	9.5E-03		1.82
PGN_1272	PG2188	*lysA*	diaminopimelate decarboxylase	Amino acid transport and metabolism	0.54	4.1E-07		
PGN_1273	PG2187	*menA*	1,4-dihydroxy-2-naphthoate octaprenyl-transferase	Coenzyme transport and metabolism	0.61	5.6E-07		
PGN_1351	PG1002	*-*	hypothetical protein	Unknown	0.66	3.3E-04		
PGN_1503	PG0465	*har*	Fur family transcriptional regulator	Inorganic ion transport and metabolism	0.15	1.8E-13		
PGN_1548	PG0419	*-*	hypothetical protein	Unknown	0.53	1.1E-03		2.59
PGN_1622	PG0339	*-*	hypothetical protein	Unknown	0.64	1.7E-03	1.83	1.27
PGN_1791	PG1858	*-*	flavodoxin FldA	Energy production and conversion	0.60	7.1E-04	15.25	1.34
PGN_2082	PG2218	*trkA*	potassium transporter peripheral membrane component	Inorganic ion transport and metabolism	0.55	4.3E-07		
PGN_2083	PG2219	*trkH*	potassium uptake protein TrkH	Inorganic ion transport and metabolism	0.66	2.8E-07		

a Results are sorted by ascending PGN_ID (locus ID in 33277).

b Predicted operons: PGN_0300-0301, PGN_0320-0321, PGN_0444-0449_b; PGN_0485-0486; PGN_0527-0531; PGN_0968-0970; PGN_1019-1021; PGN_1104-1107; PGN_1272-1273; PGN_2082-2083.

c HL - hemin-limited compared with hemin-excess as reported in Dashper *et al*. [12]. These data had p-values <0.05.

d Biofilm - biofilm compared with planktonic growth as reported in Lo *et al*. [41] and ArrayExpress E-TABM-467. These data had p-values <0.01.

**Table 9:** Validation of microarray data using qRT-PCR.

Gene	Microarray fold ratio (ECR455/33277)	qRT-PCR fold ratio (ECR455/33277)	qRT-PCR fold ratio (ECR455/ECR475)
PGN_0287	1.81	1.6	1.6
PGN_0448	0.47	0.5	0.6
PGN_1296	1	0.9	0.8
PGN_1578	1.78	1.6	1.5
PGN_2083	0.66	0.2	0.15

RNA from three biological replicates of *P. gingivalis* ATCC 33277, ECR455 and ECR475 grown in batch culture was extracted, then 800 ng RNA was reverse transcribed using random hexamers, before 0.3 ng cDNA was used as template in real time PCR with Power SYBR Green PCR master mix (Applied Biosystems) for 35 cycles. The number of copies of transcript was quantified relative to a standard curve amplified from 33277 genomic DNA for each primer pair. The mean number of transcripts from each bacterial strain was calculated for each primer pair and then used to calculate the qRT-PCR fold ratios.

### Biofilm assay

**Figure 8:**
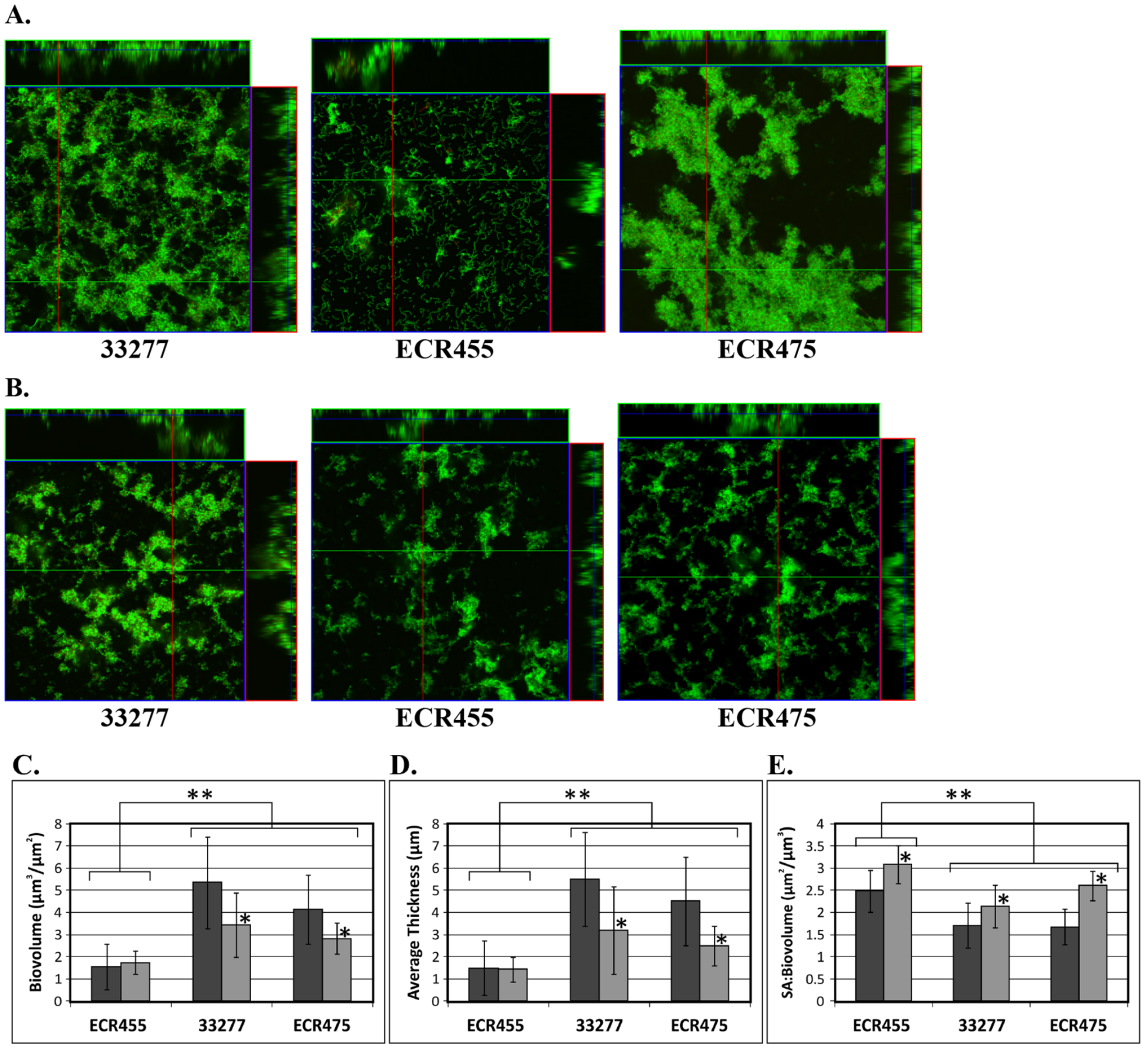
*P. gingivalis* biofilm development. Orthogonal projections of CLSM images showing a representative region of the x-y plane over the depth of the biofilm in both xz and yz dimensions of the ATCC 33277 wild-type, *har* mutant ECR455 and *har* complement ECR 475 strains grown in excess hemin (**A**) or hemin-limitation (**B**). Comparison of the Biovolume (**C**), Average Thickness (**D**) and SA:Biovolume (**E**) calculated for each strain's biofilm growth in either excess hemin (dark bars) or limited hemin (light bars) over three independent experiments. All biometric parameters analysed for the biofilms formed by ATCC 33277 and ECR475 were significantly (p<0.005) altered when hemin was limited. * indicates a significant difference in excess hemin versus limited hemin (p<0.005); ** indicates a significant difference in ECR455 biovolume, average thickness and SA:biovolume when compared to the same biometric parameters of both the ATCC 33277 and ECR475 biofilms (p<0.001).

Biometric analysis of the biofilms grown in either hemin-limited or non-limiting growth conditions showed that *P. gingivalis* ATCC 33277 wild-type and the *har* complemented ECR475 produced biofilms that were not statistically different (Fig. 8). The *har* mutant ECR455 produced biofilms that had significantly reduced biovolume and average thickness and an increased surface area (SA):biovolume ratio compared with the wild-type or ECR475 (Fig. 8). Comparison of the biofilm formed by each strain under hemin-limitation or non-limitation showed that hemin availability had no effect on the biovolume or average thickness of the biofilm formed by ECR455 whereas there was a significant reduction in the biovolume and average thickness of the wild-type and ECR475 biofilms grown under hemin limitation (Fig. 8). Planktonic growth of the three strains under the same growth conditions was similar (data not shown).

## Discussion

This study demonstrates a novel function for a Fur superfamily protein in *P. gingivalis* regulating hemin-dependent biofilm formation, a prerequisite for colonization and virulence of this bacterium within the host.

Sequence comparison indicated that Har, like other Fur superfamily members has the two -C-X-X-C- motifs associated with Zn^2+^ binding to the S1 structural site that is required for dimerization [18]. A recombinant *P. gingivalis* Har protein tightly bound one Zn^2+^ ion per monomer and oxidation of Cys residues demonstrated they were involved in metal coordination. Zn(II)Har formed a stable dimer in the absence of other divalent metal cations and therefore the results are consistent with Zn^2+^ binding to the two -C-X-X-C- motifs in the predicted S1 structural site as reported for other bacterial species [23], [24]. Mutation of Cys97 did not result in any change in Zn^2+^ binding or dimerization state of the protein indicating that this Cys residue was not a component of the S1 structural site. The need to denature Har to release Zn^2+^ is consistent with the high affinity of Zn^2+^ binding, unlike *H. pylori* Fur where EDTA-treatment alone was sufficient to remove Zn^2+^ [42].

The conserved metal binding residues (-H-X-H-) that constitute the S2 divalent metal cation sensory binding site in other Fur sequences are absent in the *P. gingivalis* Har, making it unusual amongst characterized Fur superfamily proteins (Fig. 1). *P. gingivalis* recombinant Zn(II)Har bound hemin/Fe^2+^ in a 1∶1 ratio with high affinity, indicating a novel binding site in this Fur orthologue. A Soret shift to 372 nm and 420 nm was observed upon the addition of Zn(II)Har to hemin. The Soret band shift to 420 nm has previously been used to demonstrate protein interaction via an axial Cys ligand with the central ferric ion of hemin [43]. The Soret band shift to 372 nm is typical of hemin binding to the Cys-Pro motif where pentacoordination of the central Fe^3+^ in hemin appears to be the preferred binding mechanism [44]. This motif, also known as a heme regulatory motif (HRM) features invariant Cys and Pro residues followed by a hydrophobic residue [39]. *P. gingivalis* Har has a putative HRM, -^97^C-P-^99^L- that replaces the S2 -H-X-H- motif conserved in other species, and mutation of Cys97 to Ala reduced the hemin binding affinity four-fold. This is consistent with mutation of HRMs in other hemin binding proteins such as the hemin iron sensing eukaryotic initiation factor 2α kinase (HRI) which had a five-fold decrease in hemin affinity when Cys409 of its -C-P- motif was mutated to Ser [45]. The -C-P- motif in HRI binds hemin via the coordination of Cys to the hemin iron center [45]. Given the Zn(II)Har Soret shift data and the similar effect of the Cys mutation on Zn(II)Har hemin binding compared to HRI, it is likely that Cys97 of Har binds hemin through the iron centre. This is supported by the lack of specific iron binding by the Zn(II)C97A Har protein.

Binding hemin or Fe^2+^ caused changes to the secondary structure of Zn(II)Har and Zn(II)Har150. Based on previous studies of Fur superfamily proteins conformational changes upon metal binding can have various consequences, such as inducing DNA binding [46], reversible dissociation from DNA [47] or rapid protein degradation [48].

The high pI of *P. gingivalis* Har made the study of specific DNA binding challenging due to nonspecific charge interactions. Thus we used Zn(II)Har150 with the lysine-rich tail removed for the DNA binding studies. This lysine-rich tail is only found amongst the Bacteroidetes Fur homologues. The *dnaA* promoter DNA was used as the Zn(II)Har150 binding target because in the microarray analysis, transcription of the *dnaA* gene was significantly increased in the *har* mutant ECR455 compared with wild-type, suggesting that *dnaA* is repressed by Har. The EMSA results showed that Zn(II)Har150 bound specifically to the *dnaA* promoter and that in the presence of high concentrations of hemin the binding of Har was decreased being consistent with the microarray data. These results therefore suggest that apo-Har (without its co-factor heme) is a repressor of the *dnaA* gene with upregulation of DNA replication being linked with heme availability which would better support metabolism and virulence. The repressor function of apo-Har in *P. gingivalis* is similar to the recently reported repressor function of apo-Fur in *Helicobacter pylori* [49].

The absence of the lysine-rich tail of Zn(II)Har150 may have removed some of the complexity of DNA binding regulation as the lysine-rich tail could serve as a site for lysine acetylation, a regulatory post-translational modification commonly found in eukaryotes and more recently in bacteria [50], [51]. There is precedence for this type of regulation, with *in vitro* evidence that reversible lysine acetylation modulates the DNA-binding activity of the bacterial transcriptional regulator RcsB [52].

*P. gingivalis* Har does not appear to play a role in metal ion homeostasis unlike in other bacterial species [53]–[55] as suggested by the lack of difference in the cellular content of 34 metals including iron, manganese, zinc and nickel between the *P. gingivalis har* mutant ECR455, the wild-type parental strain ATCC 33277 and the *har* complemented strain ECR475 (Table 6). DNA microarray analysis of the *P. gingivalis har* mutant ECR455 also suggested that Har may not be involved in metal ion homeostasis as only one gene known to play a role in iron/heme uptake or iron homeostasis, PGN_0558 encoding the hemophore HmuY, was differentially regulated in ECR455 (Table 7 and 8). PGN_2089 (*husC*) that was up-regulated in ECR455 may also play a role in heme uptake as it is the proposed transcriptional repressor of the HusA hemophore found only under hemin-limited conditions, however there has been no experimental characterization of HusC [30]. The results of our study are consistent with a recent report suggesting that the *P. gingivalis* Fur orthologue does not play a role in iron homeostasis [56]. Interestingly, 11 of the 35 genes that were down-regulated in ECR455 were previously seen to increase in expression under hemin-limitation (Table 8) [12] and 26 of the 35 down-regulated genes increased in expression when *P. gingivalis* was grown as a homotypic biofilm compared with planktonic growth (Table 8) [41]. This suggests that Har is a transcriptional regulator associated with heme homeostasis and biofilm formation in *P. gingivalis*. The regulation of iron homeostasis in *P. gingivalis* is likely to be complex given the importance of heme and iron to *P. gingivalis* and the complex interplay of metals in this organism [13]. James *et al*. [57] have shown that LuxS was required for a 1.5-fold increase in transcript levels of the ferrous ion transport system but negative regulators of this system have not yet been identified.

Iron availability is known to variably affect bacterial biofilm formation and development [58]–[63]. The effect of hemin availability on bacterial biofilm development is less well known but we have shown that hemin-limitation decreases homotypic *P. gingivalis* ATCC 33277 biofilm formation and development (Fig. 8) [64]. In this study we have shown that Har was required for maximal *P. gingivalis* biofilm development as demonstrated by the significant decrease in biovolume and average thickness of ECR455 biofilms under both hemin-limited and non-limited conditions compared with the ATCC 33277 wild-type and Har-complemented strain ECR475 (Fig. 8). This indicates that Har is acting as a positive regulator of biofilm formation, which is consistent with the microarray data.

The biometric analysis of the biofilms formed by the *har* mutant in hemin-limited versus non-limited growth conditions were not significantly different as they were in the wild-type indicating that Har regulated biofilm development in a hemin-responsive manner. Both ATCC 33277 and ECR475 (Har^+^) produced homotypic biofilms with significantly less biovolume under hemin-limitation compared with non-hemin limited growth conditions whilst the biovolume of the ECR455 biofilms was the same under both growth conditions. Har controls the expression of two genes important for homotypic biofilm formation, *hmuY* and *mfaI* (Table 7 and 8). Down-regulation of *hmuY* expression in ECR455 would contribute to the reduced ability of the Har mutant to form a hemin-responsive biofilm as HmuY plays a role in both hemin uptake and homotypic biofilm development in *P. gingivalis* [65], [66]. This is consistent with our previous work showing HmuY was over 2.5 times more abundant in *P. gingivalis* homotypic biofilms than in planktonic cultures [67] and *hmuY* transcription was up-regulated ten-fold under conditions of hemin-limitation in continuous culture [12]. The *mfaI* transcript encoding the *P. gingivalis* minor fimbrillin was more abundant in ECR455 and may also contribute to the reduced biovolume of the *P. gingivalis har* mutant biofilm. Although *mfaI* transcription can fluctuate over time in mature biofilms the minor fimbriae have a suppressive regulatory role on initial attachment and organization of homotypic *P. gingivalis* ATCC 33277 biofilms [68], [69].

As the Har regulon contained genes that were either positively- or negatively-regulated, it suggests that Har can act as both repressor and activator. Usually Fur orthologues act as repressor proteins, achieving positive regulation by repressing transcription of the sRNA RyhB [70]. The RNA chaperone Hfq mediates pairing between the RhyB and target mRNA, resulting in promoted degradation of mRNAs by RNase E [71]. Based on genomic sequences, *P. gingivalis* does not appear to have an Hfq orthologue, so positive and negative regulation may be achieved directly by the Har protein itself. There are examples of Fur proteins positively regulating gene expression directly [72], [73] and binding DNA in the absence of a co-factor [74]. In fact *H. pylori* Fur can function as a repressor and activator both with and without its iron cofactor [49].

## Conclusion

*P. gingivalis* is an iron and protoporphyrin IX-dependent Gram-negative bacterium that utilizes its only Fur superfamily orthologue, Har, to regulate hemin-responsive biofilm development. The ability to respond to environmental hemin and develop biofilms are key components of the *in vivo* survival and pathogenicity of *P. gingivalis*.
